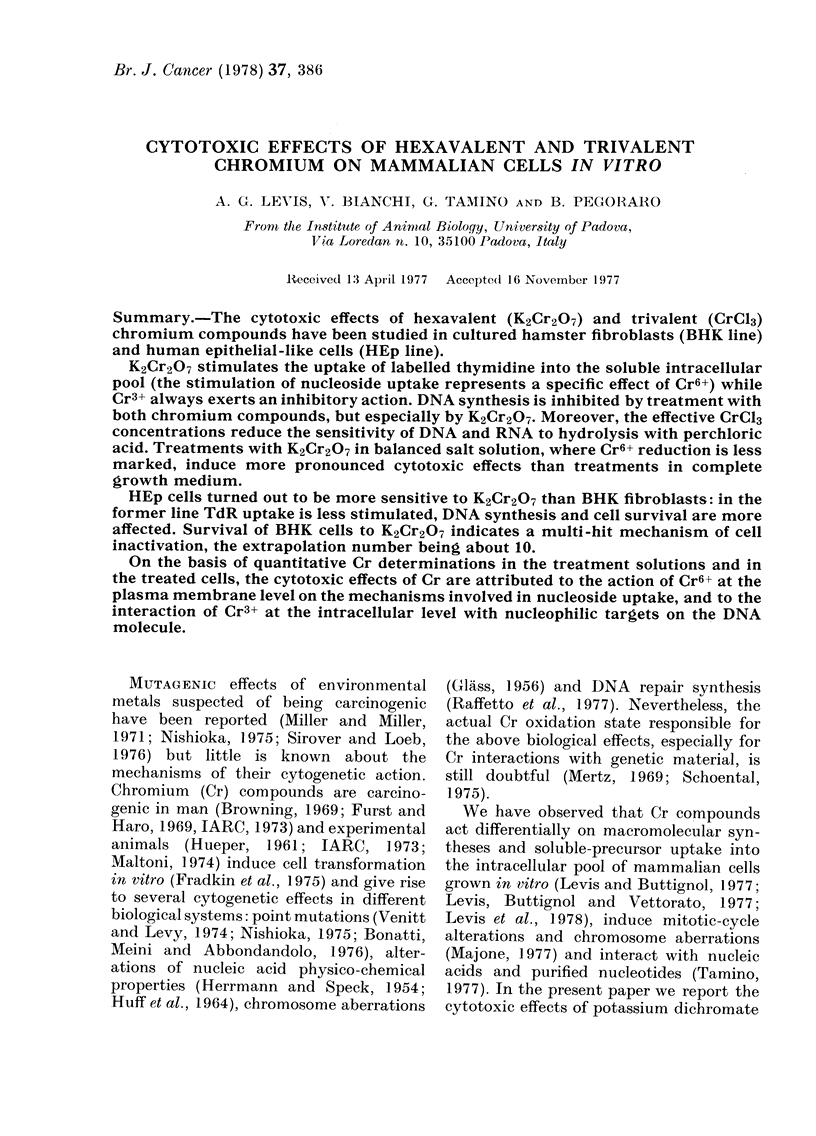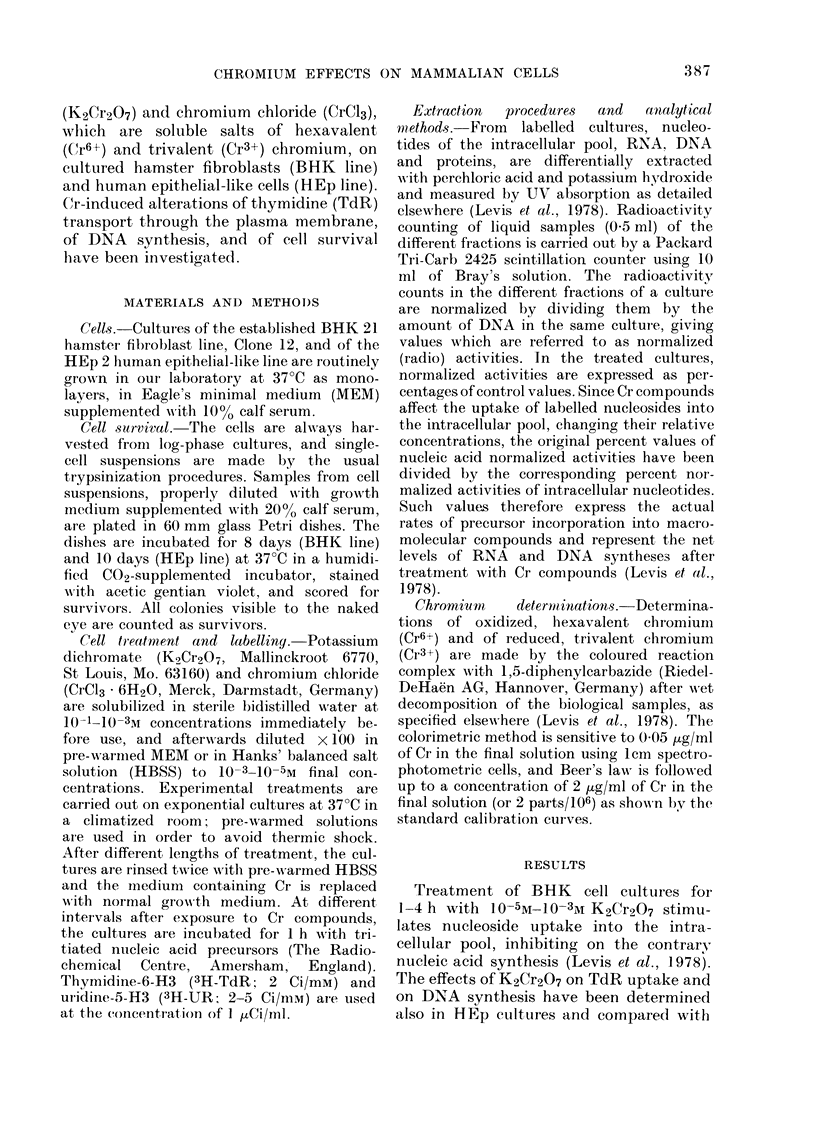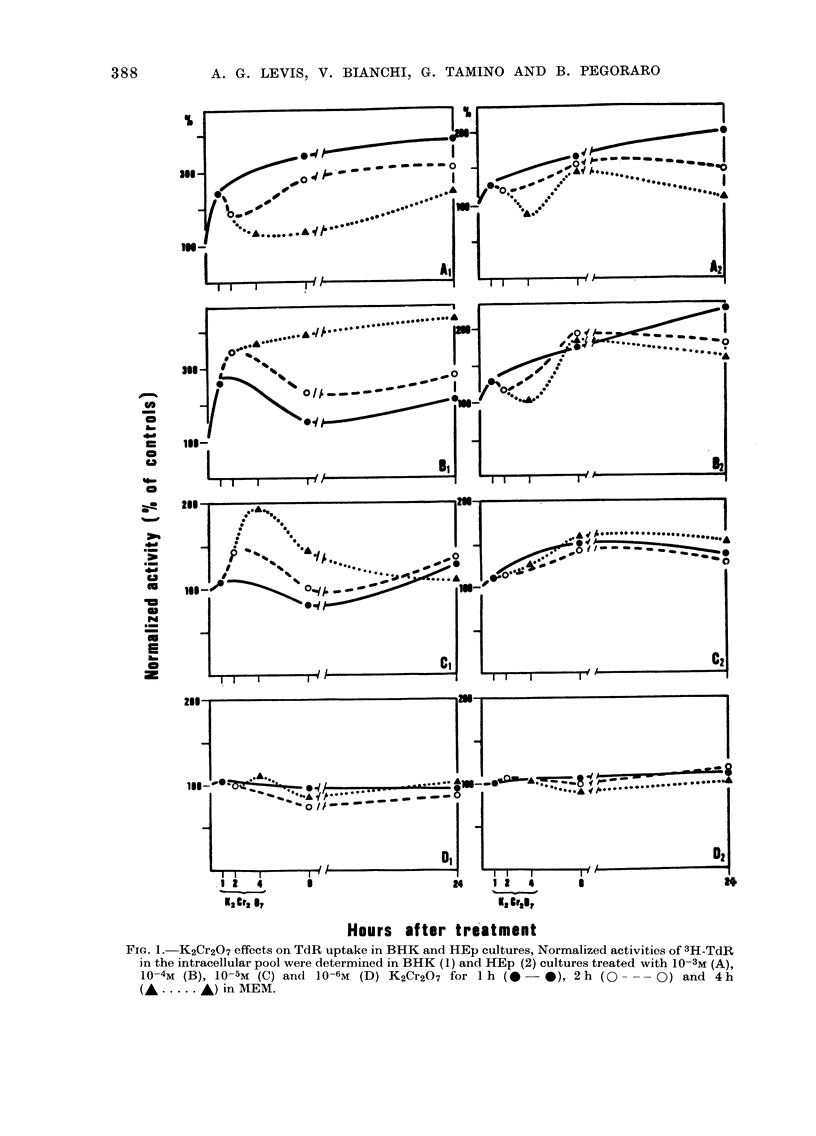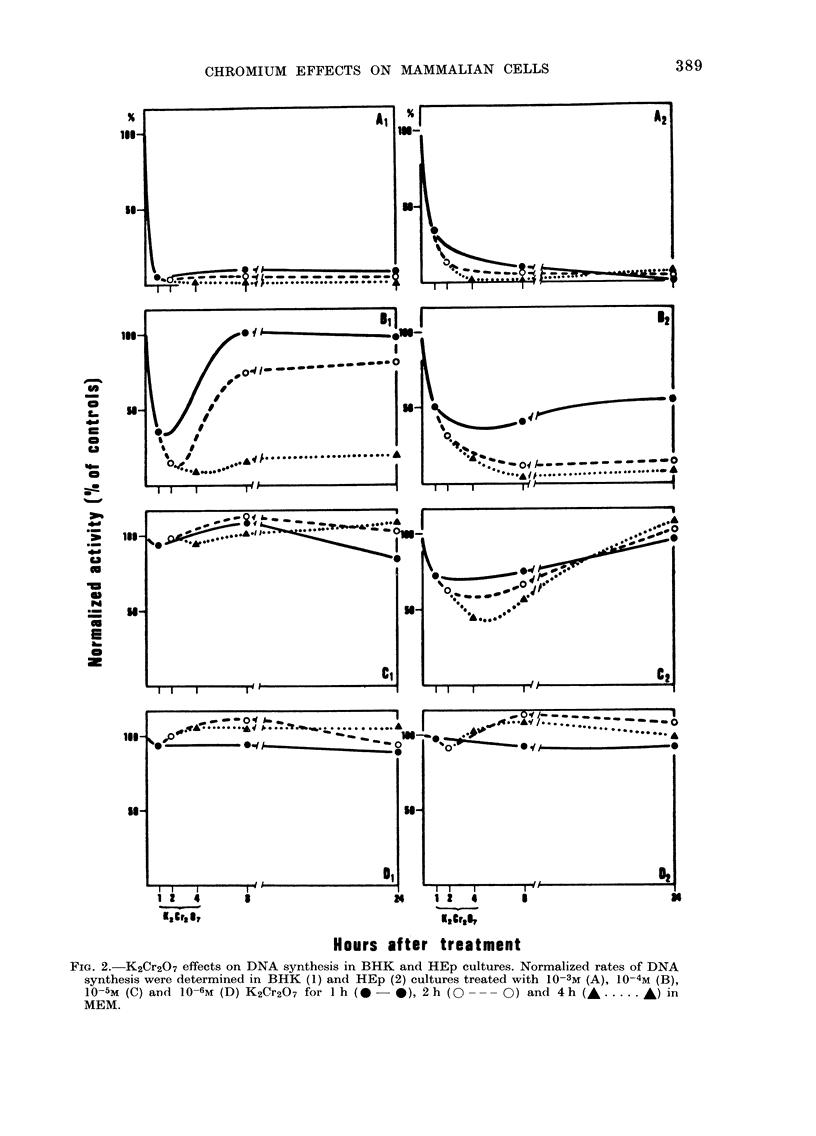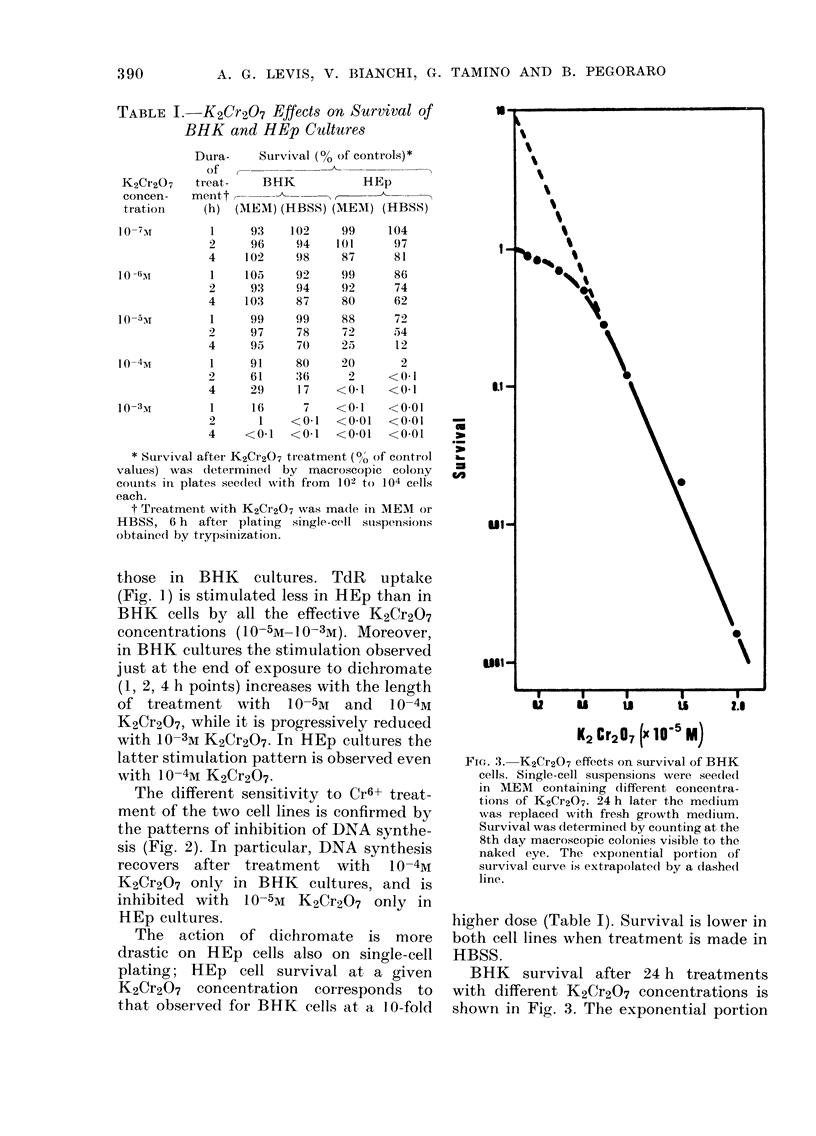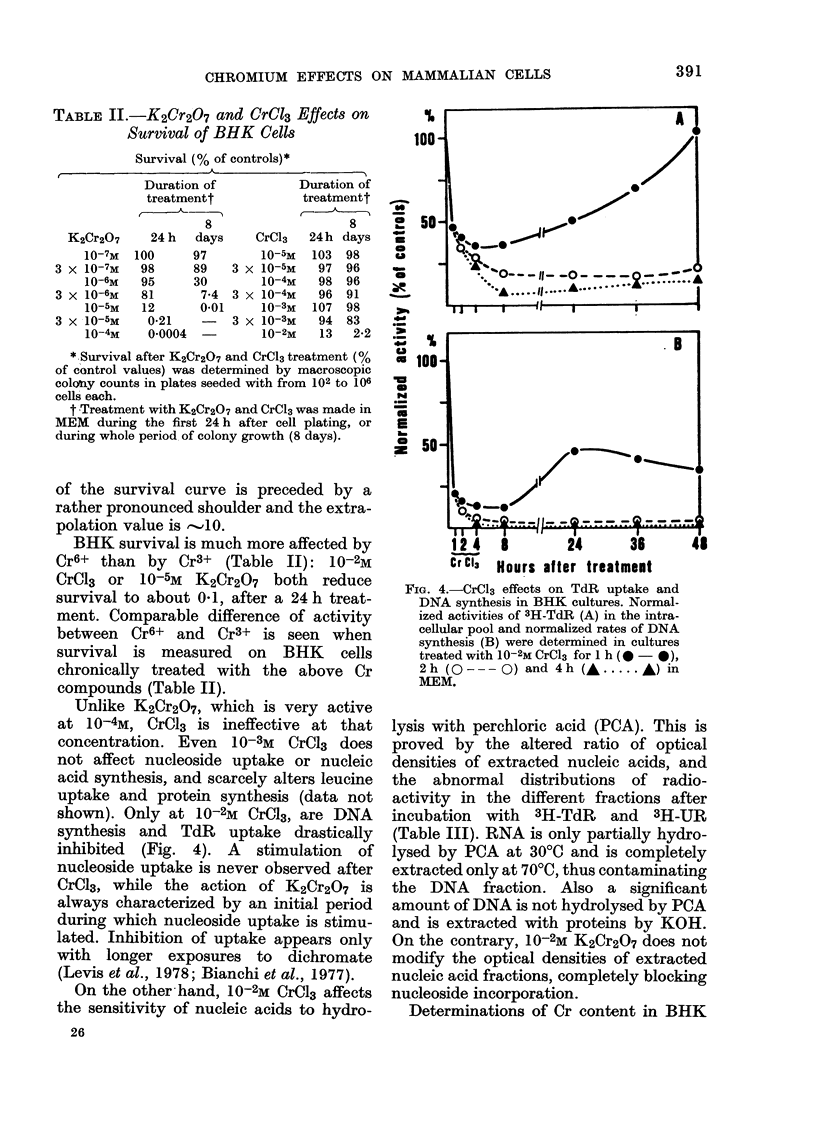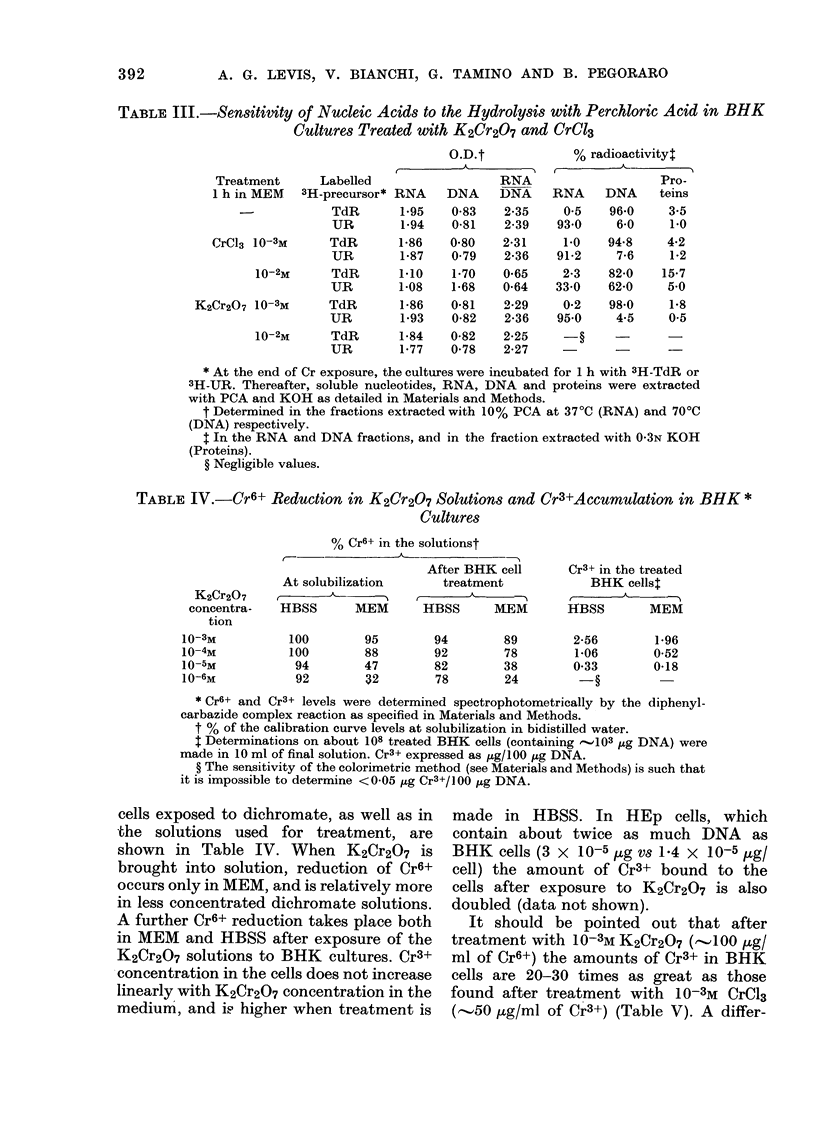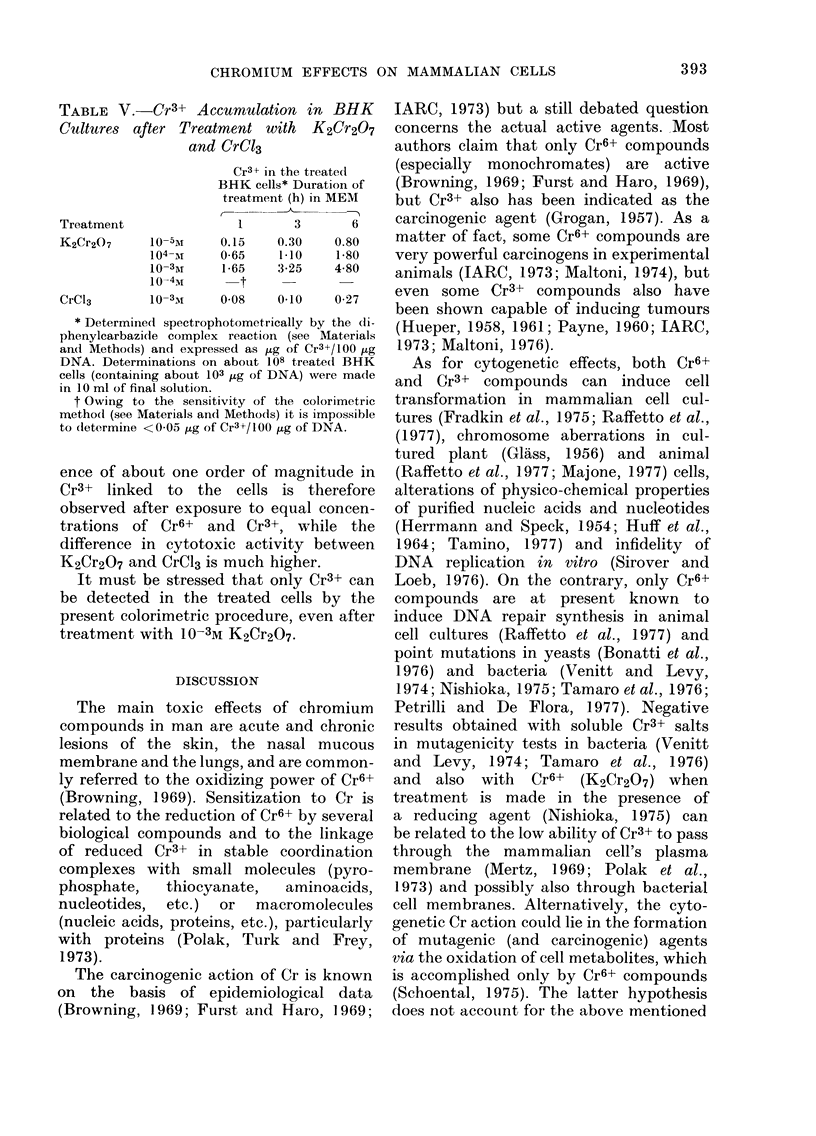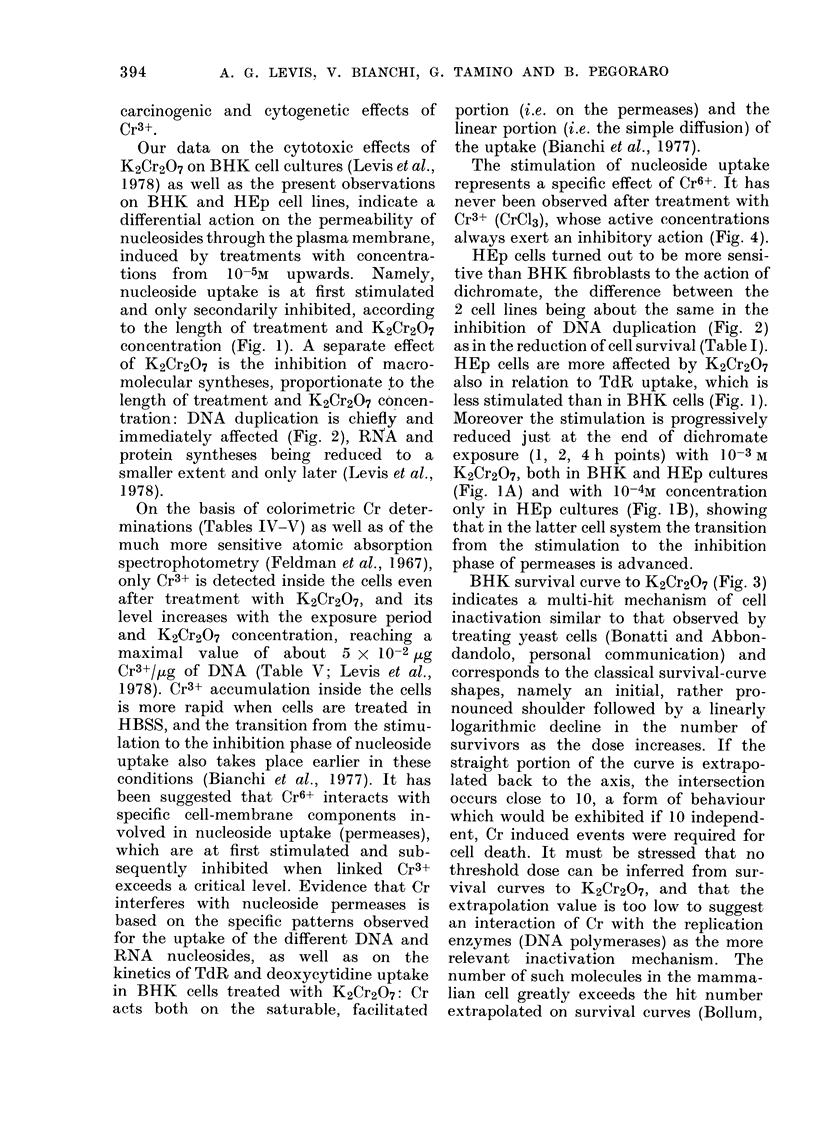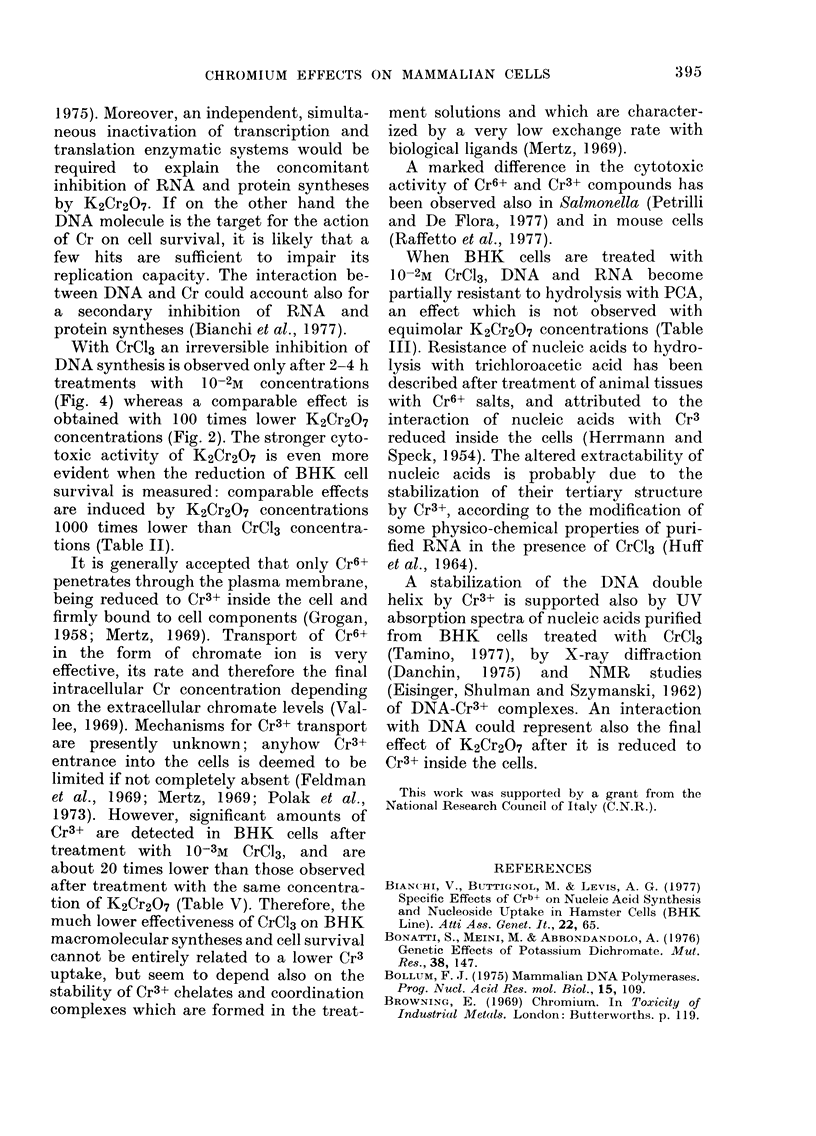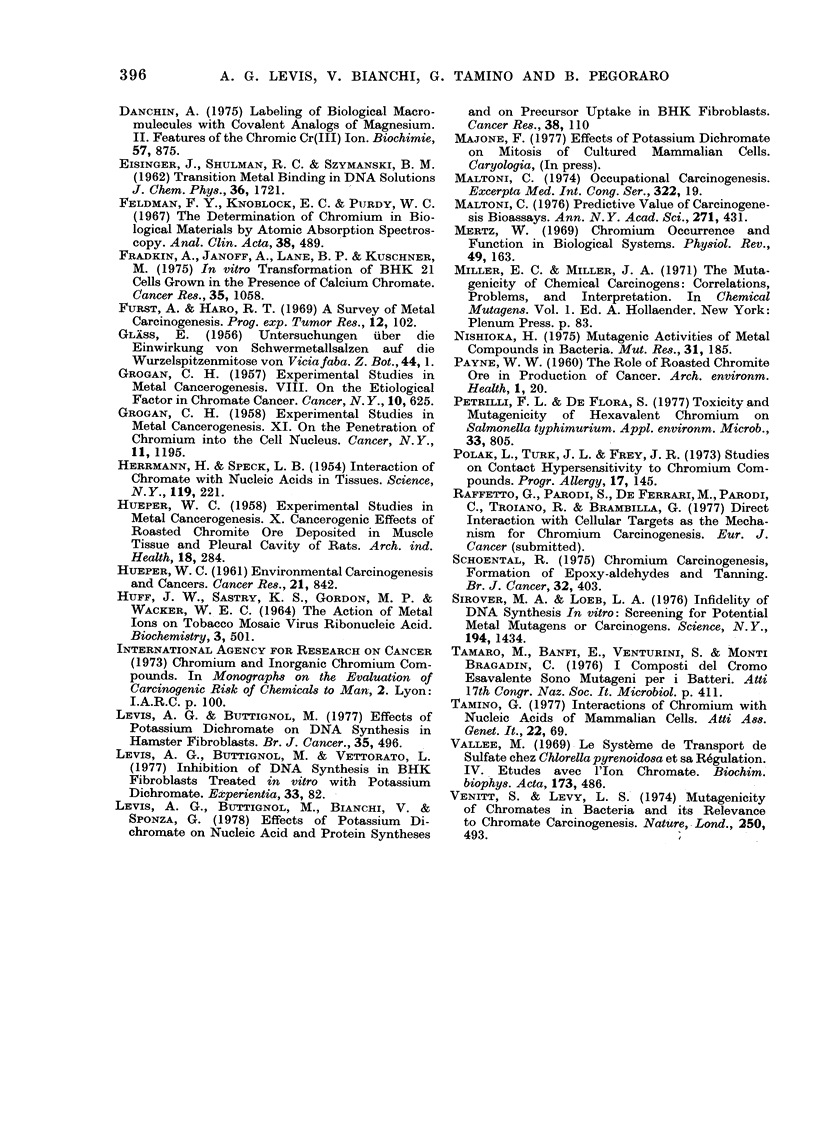# Cytotoxic Effects of Hexavalent and Trivalent Chromium on Mammalian Cells In Vitro

**DOI:** 10.1038/bjc.1978.58

**Published:** 1978-03

**Authors:** A. G. Levis, V. Bianchi, G. Tamino, B. Pegoraro

## Abstract

The cytotoxic effects of hexavalent (k_2_Cr_2_O_7_) and trivalent (CrCl_3_) chromium compounds have been studied in cultured hamster fibroblasts (BHK line) and human epithelial-like cells (HEp line).

K_2_Cr_2_O_7_ stimulates the uptake of labelled thymidine into the soluble intracellular pool (the stimulation of nucleoside uptake represents a specific effect of Cr^6+^) while Cr^3+^ always exerts an inhibitory action. DNA Synthesis is inhibited by treatment with both chromium compounds, but especially by K_2_Cr_2_O_7_. Moreover, the effective CrCl_3_ concentrations reduce the sensitivity of DNA and RNA to hydrolysis with perchloric acid. Treatments with k_2_Cr_2_O_7_ in balanced salt solution, where Cr^6+^ reduction is less marked, induce more pronounced cytotoxic effects than treatments in complete growth medium.

HEp cells turned out to be more sensitive to K_2_Cr_2_O_7_ than BHK fibroblasts: in the former line TdR uptake is less stimulated, DNA synthesis and cell survival are more affected. Survival of BHK cells to K_2_Cr_2_O_7_ indicates a multi-hit mechanism of cell inactivation, the extrapolation number being about 10.

On the basis of quantitative Cr determinations in the treatment solutions and in the treated cells, the cytotoxic effects of Cr are attributed to the action of Cr_6+_ at the plasma membrane level on the mechanisms involved in nucleoside uptake, and to the interaction of Cr^3+^ at the intracellular level with nucleophilic targets on the DNA molecule.


*Br. J. Cancer* (1978) **37**, 386

### CYTOTOXIC EFFECTS OF HEXAVALENT AND TRIVALENT CHROMIUM ON MAMMALIAN CELLS *IN VITRO*

A. G. LEVIS, V. BIANCHI, G. TAMINO AND B. PEGORARO

*From the Institute of Animal Biology, University of Padova, Via Loredan n.* 10, 35100 *Padova, Italy*

Received 13 April 1977 Accepted 16 November 1977

**Summary.—The cytotoxic effects of hexavalent ($K_2Cr_2O_7$) and trivalent ($CrCl_3$) chromium compounds have been studied in cultured hamster fibroblasts (BHK line) and human epithelial-like cells (HEp line).**

**$K_2Cr_2O_7$ stimulates the uptake of labelled thymidine into the soluble intracellular pool (the stimulation of nucleoside uptake represents a specific effect of $Cr^{6+}$) while $Cr^{3+}$ always exerts an inhibitory action. DNA synthesis is inhibited by treatment with both chromium compounds, but especially by $K_2Cr_2O_7$. Moreover, the effective $CrCl_3$ concentrations reduce the sensitivity of DNA and RNA to hydrolysis with perchloric acid. Treatments with $K_2Cr_2O_7$ in balanced salt solution, where $Cr^{6+}$ reduction is less marked, induce more pronounced cytotoxic effects than treatments in complete growth medium.**

**HEp cells turned out to be more sensitive to $K_2Cr_2O_7$ than BHK fibroblasts: in the former line TdR uptake is less stimulated, DNA synthesis and cell survival are more affected. Survival of BHK cells to $K_2Cr_2O_7$ indicates a multi-hit mechanism of cell inactivation, the extrapolation number being about 10.**

**On the basis of quantitative Cr determinations in the treatment solutions and in the treated cells, the cytotoxic effects of Cr are attributed to the action of $Cr^{6+}$ at the plasma membrane level on the mechanisms involved in nucleoside uptake, and to the interaction of $Cr^{3+}$ at the intracellular level with nucleophilic targets on the DNA molecule.**

MUTAGENIC effects of environmental metals suspected of being carcinogenic have been reported (Miller and Miller, 1971; Nishioka, 1975; Sirover and Loeb, 1976) but little is known about the mechanisms of their cytogenetic action. Chromium (Cr) compounds are carcinogenic in man (Browning, 1969; Furst and Haro, 1969, IARC, 1973) and experimental animals (Hueper, 1961; IARC, 1973; Maltoni, 1974) induce cell transformation *in vitro* (Fradkin *et al.*, 1975) and give rise to several cytogenetic effects in different biological systems: point mutations (Venitt and Levy, 1974; Nishioka, 1975; Bonatti, Meini and Abbondandolo, 1976), alterations of nucleic acid physico-chemical properties (Herrmann and Speck, 1954; Huff *et al.*, 1964), chromosome aberrations (Gläss, 1956) and DNA repair synthesis (Raffetto *et al.*, 1977). Nevertheless, the actual Cr oxidation state responsible for the above biological effects, especially for Cr interactions with genetic material, is still doubtful (Mertz, 1969; Schoental, 1975).

We have observed that Cr compounds act differentially on macromolecular syntheses and soluble-precursor uptake into the intracellular pool of mammalian cells grown *in vitro* (Levis and Buttignol, 1977; Levis, Buttignol and Vettorato, 1977; Levis *et al.*, 1978), induce mitotic-cycle alterations and chromosome aberrations (Majone, 1977) and interact with nucleic acids and purified nucleotides (Tamino, 1977). In the present paper we report the cytotoxic effects of potassium dichromate

($K_2Cr_2O_7$) and chromium chloride ($CrCl_3$), which are soluble salts of hexavalent ($Cr^{6+}$) and trivalent ($Cr^{3+}$) chromium, on cultured hamster fibroblasts (BHK line) and human epithelial-like cells (HEp line). Cr-induced alterations of thymidine (TdR) transport through the plasma membrane, of DNA synthesis, and of cell survival have been investigated.

### MATERIALS AND METHODS

*Cells.*—Cultures of the established BHK 21 hamster fibroblast line, Clone 12, and of the HEp 2 human epithelial-like line are routinely grown in our laboratory at 37°C as monolayers, in Eagle's minimal medium (MEM) supplemented with 10% calf serum.

*Cell survival.*—The cells are always harvested from log-phase cultures, and single-cell suspensions are made by the usual trypsinization procedures. Samples from cell suspensions, properly diluted with growth medium supplemented with 20% calf serum, are plated in 60 mm glass Petri dishes. The dishes are incubated for 8 days (BHK line) and 10 days (HEp line) at 37°C in a humidified $CO_2$-supplemented incubator, stained with acetic gentian violet, and scored for survivors. All colonies visible to the naked eye are counted as survivors.

*Cell treatment and labelling.*—Potassium dichromate ($K_2Cr_2O_7$, Mallinckroot 6770, St Louis, Mo. 63160) and chromium chloride ($CrCl_3 \cdot 6H_2O$, Merck, Darmstadt, Germany) are solubilized in sterile bidistilled water at $10^{-1}$–$10^{-3}$M concentrations immediately before use, and afterwards diluted $\times 100$ in pre-warmed MEM or in Hanks' balanced salt solution (HBSS) to $10^{-3}$–$10^{-5}$M final concentrations. Experimental treatments are carried out on exponential cultures at 37°C in a climatized room; pre-warmed solutions are used in order to avoid thermic shock. After different lengths of treatment, the cultures are rinsed twice with pre-warmed HBSS and the medium containing Cr is replaced with normal growth medium. At different intervals after exposure to Cr compounds, the cultures are incubated for 1 h with tritiated nucleic acid precursors (The Radiochemical Centre, Amersham, England). Thymidine-6-H3 ($^3$H-TdR; 2 Ci/mM) and uridine-5-H3 ($^3$H-UR; 2–5 Ci/mM) are used at the concentration of 1 $\mu$Ci/ml.

*Extraction procedures and analytical methods.*—From labelled cultures, nucleotides of the intracellular pool, RNA, DNA and proteins, are differentially extracted with perchloric acid and potassium hydroxide and measured by UV absorption as detailed elsewhere (Levis *et al.*, 1978). Radioactivity counting of liquid samples (0·5 ml) of the different fractions is carried out by a Packard Tri-Carb 2425 scintillation counter using 10 ml of Bray's solution. The radioactivity counts in the different fractions of a culture are normalized by dividing them by the amount of DNA in the same culture, giving values which are referred to as normalized (radio) activities. In the treated cultures, normalized activities are expressed as percentages of control values. Since Cr compounds affect the uptake of labelled nucleosides into the intracellular pool, changing their relative concentrations, the original percent values of nucleic acid normalized activities have been divided by the corresponding percent normalized activities of intracellular nucleotides. Such values therefore express the actual rates of precursor incorporation into macromolecular compounds and represent the net levels of RNA and DNA syntheses after treatment with Cr compounds (Levis *et al.*, 1978).

*Chromium determinations.*—Determinations of oxidized, hexavalent chromium ($Cr^{6+}$) and of reduced, trivalent chromium ($Cr^{3+}$) are made by the coloured reaction complex with 1,5-diphenylcarbazide (Riedel-DeHaën AG, Hannover, Germany) after wet decomposition of the biological samples, as specified elsewhere (Levis *et al.*, 1978). The colorimetric method is sensitive to 0·05 $\mu$g/ml of Cr in the final solution using 1cm spectrophotometric cells, and Beer's law is followed up to a concentration of 2 $\mu$g/ml of Cr in the final solution (or 2 parts/$10^6$) as shown by the standard calibration curves.

### RESULTS

Treatment of BHK cell cultures for 1–4 h with $10^{-5}$M–$10^{-3}$M $K_2Cr_2O_7$ stimulates nucleoside uptake into the intracellular pool, inhibiting on the contrary nucleic acid synthesis (Levis *et al.*, 1978). The effects of $K_2Cr_2O_7$ on TdR uptake and on DNA synthesis have been determined also in HEp cultures and compared with

FIG. 1.—$K_2Cr_2O_7$ effects on TdR uptake in BHK and HEp cultures, Normalized activities of $^3$H-TdR in the intracellular pool were determined in BHK (1) and HEp (2) cultures treated with $10^{-3}$M (A), $10^{-4}$M (B), $10^{-5}$M (C) and $10^{-6}$M (D) $K_2Cr_2O_7$ for 1 h (● — ●), 2 h (○ - - - ○) and 4 h (▲ . . . . . ▲) in MEM.

FIG. 2.—$K_2Cr_2O_7$ effects on DNA synthesis in BHK and HEp cultures. Normalized rates of DNA synthesis were determined in BHK (1) and HEp (2) cultures treated with $10^{-3}$M (A), $10^{-4}$M (B), $10^{-5}$M (C) and $10^{-6}$M (D) $K_2Cr_2O_7$ for 1 h (● — ●), 2 h (○ - - - ○) and 4 h (▲ . . . . . ▲) in MEM.

TABLE I.—*$K_2Cr_2O_7$ Effects on Survival of BHK and HEp Cultures*

| $K_2Cr_2O_7$ concentration | Duration of treatment† (h) | Survival (% of controls)* BHK (MEM) | BHK (HBSS) | HEp (MEM) | HEp (HBSS) |
|---|---|---|---|---|---|
| $10^{-7}$M | 1 | 93 | 102 | 99 | 104 |
| | 2 | 96 | 94 | 101 | 97 |
| | 4 | 102 | 98 | 87 | 81 |
| $10^{-6}$M | 1 | 105 | 92 | 99 | 86 |
| | 2 | 93 | 94 | 92 | 74 |
| | 4 | 103 | 87 | 80 | 62 |
| $10^{-5}$M | 1 | 99 | 99 | 88 | 72 |
| | 2 | 97 | 78 | 72 | 54 |
| | 4 | 95 | 70 | 25 | 12 |
| $10^{-4}$M | 1 | 91 | 80 | 20 | 2 |
| | 2 | 61 | 36 | 2 | <0·1 |
| | 4 | 29 | 17 | <0·1 | <0·1 |
| $10^{-3}$M | 1 | 16 | 7 | <0·1 | <0·01 |
| | 2 | 1 | <0·1 | <0·01 | <0·01 |
| | 4 | <0·1 | <0·1 | <0·01 | <0·01 |

* Survival after $K_2Cr_2O_7$ treatment (% of control values) was determined by macroscopic colony counts in plates seeded with from $10^2$ to $10^4$ cells each.

† Treatment with $K_2Cr_2O_7$ was made in MEM or HBSS, 6 h after plating single-cell suspensions obtained by trypsinization.

those in BHK cultures. TdR uptake (Fig. 1) is stimulated less in HEp than in BHK cells by all the effective $K_2Cr_2O_7$ concentrations ($10^{-5}$M–$10^{-3}$M). Moreover, in BHK cultures the stimulation observed just at the end of exposure to dichromate (1, 2, 4 h points) increases with the length of treatment with $10^{-5}$M and $10^{-4}$M $K_2Cr_2O_7$, while it is progressively reduced with $10^{-3}$M $K_2Cr_2O_7$. In HEp cultures the latter stimulation pattern is observed even with $10^{-4}$M $K_2Cr_2O_7$.

The different sensitivity to $Cr^{6+}$ treatment of the two cell lines is confirmed by the patterns of inhibition of DNA synthesis (Fig. 2). In particular, DNA synthesis recovers after treatment with $10^{-4}$M $K_2Cr_2O_7$ only in BHK cultures, and is inhibited with $10^{-5}$M $K_2Cr_2O_7$ only in HEp cultures.

The action of dichromate is more drastic on HEp cells also on single-cell plating; HEp cell survival at a given $K_2Cr_2O_7$ concentration corresponds to that observed for BHK cells at a 10-fold

FIG. 3.—$K_2Cr_2O_7$ effects on survival of BHK cells. Single-cell suspensions were seeded in MEM containing different concentrations of $K_2Cr_2O_7$. 24 h later the medium was replaced with fresh growth medium. Survival was determined by counting at the 8th day macroscopic colonies visible to the naked eye. The exponential portion of survival curve is extrapolated by a dashed line.

higher dose (Table I). Survival is lower in both cell lines when treatment is made in HBSS.

BHK survival after 24 h treatments with different $K_2Cr_2O_7$ concentrations is shown in Fig. 3. The exponential portion

TABLE II.—*$K_2Cr_2O_7$ and $CrCl_3$ Effects on Survival of BHK Cells*

| | Survival (% of controls)* | | | | |
|---|---|---|---|---|---|
| | Duration of treatment† | | | Duration of treatment† | |
| $K_2Cr_2O_7$ | 24 h | 8 days | $CrCl_3$ | 24 h | 8 days |
| $10^{-7}$M | 100 | 97 | $10^{-5}$M | 103 | 98 |
| $3 \times 10^{-7}$M | 98 | 89 | $3 \times 10^{-5}$M | 97 | 96 |
| $10^{-6}$M | 95 | 30 | $10^{-4}$M | 98 | 96 |
| $3 \times 10^{-6}$M | 81 | 7·4 | $3 \times 10^{-4}$M | 96 | 91 |
| $10^{-5}$M | 12 | 0·01 | $10^{-3}$M | 107 | 98 |
| $3 \times 10^{-5}$M | 0·21 | — | $3 \times 10^{-3}$M | 94 | 83 |
| $10^{-4}$M | 0·0004 | — | $10^{-2}$M | 13 | 2·2 |

* Survival after $K_2Cr_2O_7$ and $CrCl_3$ treatment (% of control values) was determined by macroscopic colony counts in plates seeded with from $10^2$ to $10^6$ cells each.

† Treatment with $K_2Cr_2O_7$ and $CrCl_3$ was made in MEM during the first 24 h after cell plating, or during whole period of colony growth (8 days).

of the survival curve is preceded by a rather pronounced shoulder and the extrapolation value is ~10.

BHK survival is much more affected by $Cr^{6+}$ than by $Cr^{3+}$ (Table II): $10^{-2}$M $CrCl_3$ or $10^{-5}$M $K_2Cr_2O_7$ both reduce survival to about 0·1, after a 24 h treatment. Comparable difference of activity between $Cr^{6+}$ and $Cr^{3+}$ is seen when survival is measured on BHK cells chronically treated with the above Cr compounds (Table II).

Unlike $K_2Cr_2O_7$, which is very active at $10^{-4}$M, $CrCl_3$ is ineffective at that concentration. Even $10^{-3}$M $CrCl_3$ does not affect nucleoside uptake or nucleic acid synthesis, and scarcely alters leucine uptake and protein synthesis (data not shown). Only at $10^{-2}$M $CrCl_3$, are DNA synthesis and TdR uptake drastically inhibited (Fig. 4). A stimulation of nucleoside uptake is never observed after $CrCl_3$, while the action of $K_2Cr_2O_7$ is always characterized by an initial period during which nucleoside uptake is stimulated. Inhibition of uptake appears only with longer exposures to dichromate (Levis *et al.*, 1978; Bianchi *et al.*, 1977).

On the other hand, $10^{-2}$M $CrCl_3$ affects the sensitivity of nucleic acids to hydro-

FIG. 4.—$CrCl_3$ effects on TdR uptake and DNA synthesis in BHK cultures. Normalized activities of $^3$H-TdR (A) in the intracellular pool and normalized rates of DNA synthesis (B) were determined in cultures treated with $10^{-2}$M $CrCl_3$ for 1 h (● — ●), 2 h (○ - - - ○) and 4 h (▲ . . . . . ▲) in MEM.

lysis with perchloric acid (PCA). This is proved by the altered ratio of optical densities of extracted nucleic acids, and the abnormal distributions of radioactivity in the different fractions after incubation with $^3$H-TdR and $^3$H-UR (Table III). RNA is only partially hydrolysed by PCA at 30°C and is completely extracted only at 70°C, thus contaminating the DNA fraction. Also a significant amount of DNA is not hydrolysed by PCA and is extracted with proteins by KOH. On the contrary, $10^{-2}$M $K_2Cr_2O_7$ does not modify the optical densities of extracted nucleic acid fractions, completely blocking nucleoside incorporation.

Determinations of Cr content in BHK

TABLE III.—*Sensitivity of Nucleic Acids to the Hydrolysis with Perchloric Acid in BHK Cultures Treated with $K_2Cr_2O_7$ and $CrCl_3$*

| Treatment 1 h in MEM | Labelled $^3$H-precursor* | O.D.† RNA | O.D.† DNA | O.D.† RNA/DNA | % radioactivity‡ RNA | % radioactivity‡ DNA | % radioactivity‡ Proteins |
|---|---|---|---|---|---|---|---|
| — | TdR | 1·95 | 0·83 | 2·35 | 0·5 | 96·0 | 3·5 |
| | UR | 1·94 | 0·81 | 2·39 | 93·0 | 6·0 | 1·0 |
| $CrCl_3$ $10^{-3}$M | TdR | 1·86 | 0·80 | 2·31 | 1·0 | 94·8 | 4·2 |
| | UR | 1·87 | 0·79 | 2·36 | 91·2 | 7·6 | 1·2 |
| $10^{-2}$M | TdR | 1·10 | 1·70 | 0·65 | 2·3 | 82·0 | 15·7 |
| | UR | 1·08 | 1·68 | 0·64 | 33·0 | 62·0 | 5·0 |
| $K_2Cr_2O_7$ $10^{-3}$M | TdR | 1·86 | 0·81 | 2·29 | 0·2 | 98·0 | 1·8 |
| | UR | 1·93 | 0·82 | 2·36 | 95·0 | 4·5 | 0·5 |
| $10^{-2}$M | TdR | 1·84 | 0·82 | 2·25 | —§ | — | — |
| | UR | 1·77 | 0·78 | 2·27 | — | — | — |

* At the end of Cr exposure, the cultures were incubated for 1 h with $^3$H-TdR or $^3$H-UR. Thereafter, soluble nucleotides, RNA, DNA and proteins were extracted with PCA and KOH as detailed in Materials and Methods.

† Determined in the fractions extracted with 10% PCA at 37°C (RNA) and 70°C (DNA) respectively.

‡ In the RNA and DNA fractions, and in the fraction extracted with 0·3N KOH (Proteins).

§ Negligible values.

TABLE IV.—*$Cr^{6+}$ Reduction in $K_2Cr_2O_7$ Solutions and $Cr^{3+}$Accumulation in BHK* Cultures*

| $K_2Cr_2O_7$ concentration | % $Cr^{6+}$ in the solutions† At solubilization HBSS | At solubilization MEM | After BHK cell treatment HBSS | After BHK cell treatment MEM | $Cr^{3+}$ in the treated BHK cells‡ HBSS | $Cr^{3+}$ in the treated BHK cells‡ MEM |
|---|---|---|---|---|---|---|
| $10^{-3}$M | 100 | 95 | 94 | 89 | 2·56 | 1·96 |
| $10^{-4}$M | 100 | 88 | 92 | 78 | 1·06 | 0·52 |
| $10^{-5}$M | 94 | 47 | 82 | 38 | 0·33 | 0·18 |
| $10^{-6}$M | 92 | 32 | 78 | 24 | —§ | — |

* $Cr^{6+}$ and $Cr^{3+}$ levels were determined spectrophotometrically by the diphenylcarbazide complex reaction as specified in Materials and Methods.

† % of the calibration curve levels at solubilization in bidistilled water.

‡ Determinations on about $10^8$ treated BHK cells (containing $\sim 10^3$ $\mu$g DNA) were made in 10 ml of final solution. $Cr^{3+}$ expressed as $\mu$g/100 $\mu$g DNA.

§ The sensitivity of the colorimetric method (see Materials and Methods) is such that it is impossible to determine $<0{\cdot}05$ $\mu$g $Cr^{3+}$/100 $\mu$g DNA.

cells exposed to dichromate, as well as in the solutions used for treatment, are shown in Table IV. When $K_2Cr_2O_7$ is brought into solution, reduction of $Cr^{6+}$ occurs only in MEM, and is relatively more in less concentrated dichromate solutions. A further $Cr^{6+}$ reduction takes place both in MEM and HBSS after exposure of the $K_2Cr_2O_7$ solutions to BHK cultures. $Cr^{3+}$ concentration in the cells does not increase linearly with $K_2Cr_2O_7$ concentration in the medium, and is higher when treatment is made in HBSS. In HEp cells, which contain about twice as much DNA as BHK cells ($3 \times 10^{-5}$ $\mu$g *vs* $1{\cdot}4 \times 10^{-5}$ $\mu$g/cell) the amount of $Cr^{3+}$ bound to the cells after exposure to $K_2Cr_2O_7$ is also doubled (data not shown).

It should be pointed out that after treatment with $10^{-3}$M $K_2Cr_2O_7$ ($\sim$100 $\mu$g/ml of $Cr^{6+}$) the amounts of $Cr^{3+}$ in BHK cells are 20–30 times as great as those found after treatment with $10^{-3}$M $CrCl_3$ ($\sim$50 $\mu$g/ml of $Cr^{3+}$) (Table V). A differ-

TABLE V.—*$Cr^{3+}$ Accumulation in BHK Cultures after Treatment with $K_2Cr_2O_7$ and $CrCl_3$*

| Treatment | | $Cr^{3+}$ in the treated BHK cells* Duration of treatment (h) in MEM | | |
|---|---|---|---|---|
| | | 1 | 3 | 6 |
| $K_2Cr_2O_7$ | $10^{-5}$M | 0.15 | 0.30 | 0.80 |
| | $10^{4-}$M | 0·65 | 1·10 | 1·80 |
| | $10^{-3}$M | 1·65 | 3·25 | 4·80 |
| | $10^{-4}$M | —† | — | — |
| $CrCl_3$ | $10^{-3}$M | 0·08 | 0·10 | 0·27 |

* Determined spectrophotometrically by the diphenylcarbazide complex reaction (see Materials and Methods) and expressed as μg of $Cr^{3+}$/100 μg DNA. Determinations on about $10^8$ treated BHK cells (containing about $10^3$ μg of DNA) were made in 10 ml of final solution.

† Owing to the sensitivity of the colorimetric method (see Materials and Methods) it is impossible to determine <0·05 μg of $Cr^{3+}$/100 μg of DNA.

ence of about one order of magnitude in $Cr^{3+}$ linked to the cells is therefore observed after exposure to equal concentrations of $Cr^{6+}$ and $Cr^{3+}$, while the difference in cytotoxic activity between $K_2Cr_2O_7$ and $CrCl_3$ is much higher.

It must be stressed that only $Cr^{3+}$ can be detected in the treated cells by the present colorimetric procedure, even after treatment with $10^{-3}$M $K_2Cr_2O_7$.

### DISCUSSION

The main toxic effects of chromium compounds in man are acute and chronic lesions of the skin, the nasal mucous membrane and the lungs, and are commonly referred to the oxidizing power of $Cr^{6+}$ (Browning, 1969). Sensitization to Cr is related to the reduction of $Cr^{6+}$ by several biological compounds and to the linkage of reduced $Cr^{3+}$ in stable coordination complexes with small molecules (pyrophosphate, thiocyanate, aminoacids, nucleotides, etc.) or macromolecules (nucleic acids, proteins, etc.), particularly with proteins (Polak, Turk and Frey, 1973).

The carcinogenic action of Cr is known on the basis of epidemiological data (Browning, 1969; Furst and Haro, 1969; IARC, 1973) but a still debated question concerns the actual active agents. Most authors claim that only $Cr^{6+}$ compounds (especially monochromates) are active (Browning, 1969; Furst and Haro, 1969), but $Cr^{3+}$ also has been indicated as the carcinogenic agent (Grogan, 1957). As a matter of fact, some $Cr^{6+}$ compounds are very powerful carcinogens in experimental animals (IARC, 1973; Maltoni, 1974), but even some $Cr^{3+}$ compounds also have been shown capable of inducing tumours (Hueper, 1958, 1961; Payne, 1960; IARC, 1973; Maltoni, 1976).

As for cytogenetic effects, both $Cr^{6+}$ and $Cr^{3+}$ compounds can induce cell transformation in mammalian cell cultures (Fradkin *et al.*, 1975; Raffetto *et al.*, (1977), chromosome aberrations in cultured plant (Gläss, 1956) and animal (Raffetto *et al.*, 1977; Majone, 1977) cells, alterations of physico-chemical properties of purified nucleic acids and nucleotides (Herrmann and Speck, 1954; Huff *et al.*, 1964; Tamino, 1977) and infidelity of DNA replication *in vitro* (Sirover and Loeb, 1976). On the contrary, only $Cr^{6+}$ compounds are at present known to induce DNA repair synthesis in animal cell cultures (Raffetto *et al.*, 1977) and point mutations in yeasts (Bonatti *et al.*, 1976) and bacteria (Venitt and Levy, 1974; Nishioka, 1975; Tamaro *et al.*, 1976; Petrilli and De Flora, 1977). Negative results obtained with soluble $Cr^{3+}$ salts in mutagenicity tests in bacteria (Venitt and Levy, 1974; Tamaro *et al.*, 1976) and also with $Cr^{6+}$ ($K_2Cr_2O_7$) when treatment is made in the presence of a reducing agent (Nishioka, 1975) can be related to the low ability of $Cr^{3+}$ to pass through the mammalian cell's plasma membrane (Mertz, 1969; Polak *et al.*, 1973) and possibly also through bacterial cell membranes. Alternatively, the cytogenetic Cr action could lie in the formation of mutagenic (and carcinogenic) agents *via* the oxidation of cell metabolites, which is accomplished only by $Cr^{6+}$ compounds (Schoental, 1975). The latter hypothesis does not account for the above mentioned carcinogenic and cytogenetic effects of $Cr^{3+}$.

Our data on the cytotoxic effects of $K_2Cr_2O_7$ on BHK cell cultures (Levis *et al.*, 1978) as well as the present observations on BHK and HEp cell lines, indicate a differential action on the permeability of nucleosides through the plasma membrane, induced by treatments with concentrations from $10^{-5}$M upwards. Namely, nucleoside uptake is at first stimulated and only secondarily inhibited, according to the length of treatment and $K_2Cr_2O_7$ concentration (Fig. 1). A separate effect of $K_2Cr_2O_7$ is the inhibition of macromolecular syntheses, proportionate to the length of treatment and $K_2Cr_2O_7$ concentration: DNA duplication is chiefly and immediately affected (Fig. 2), RNA and protein syntheses being reduced to a smaller extent and only later (Levis *et al.*, 1978).

On the basis of colorimetric Cr determinations (Tables IV–V) as well as of the much more sensitive atomic absorption spectrophotometry (Feldman *et al.*, 1967), only $Cr^{3+}$ is detected inside the cells even after treatment with $K_2Cr_2O_7$, and its level increases with the exposure period and $K_2Cr_2O_7$ concentration, reaching a maximal value of about $5 \times 10^{-2}$ $\mu$g $Cr^{3+}/\mu$g of DNA (Table V; Levis *et al.*, 1978). $Cr^{3+}$ accumulation inside the cells is more rapid when cells are treated in HBSS, and the transition from the stimulation to the inhibition phase of nucleoside uptake also takes place earlier in these conditions (Bianchi *et al.*, 1977). It has been suggested that $Cr^{6+}$ interacts with specific cell-membrane components involved in nucleoside uptake (permeases), which are at first stimulated and subsequently inhibited when linked $Cr^{3+}$ exceeds a critical level. Evidence that Cr interferes with nucleoside permeases is based on the specific patterns observed for the uptake of the different DNA and RNA nucleosides, as well as on the kinetics of TdR and deoxycytidine uptake in BHK cells treated with $K_2Cr_2O_7$: Cr acts both on the saturable, facilitated portion (*i.e.* on the permeases) and the linear portion (*i.e.* the simple diffusion) of the uptake (Bianchi *et al.*, 1977).

The stimulation of nucleoside uptake represents a specific effect of $Cr^{6+}$. It has never been observed after treatment with $Cr^{3+}$ ($CrCl_3$), whose active concentrations always exert an inhibitory action (Fig. 4).

HEp cells turned out to be more sensitive than BHK fibroblasts to the action of dichromate, the difference between the 2 cell lines being about the same in the inhibition of DNA duplication (Fig. 2) as in the reduction of cell survival (Table I). HEp cells are more affected by $K_2Cr_2O_7$ also in relation to TdR uptake, which is less stimulated than in BHK cells (Fig. 1). Moreover the stimulation is progressively reduced just at the end of dichromate exposure (1, 2, 4 h points) with $10^{-3}$ M $K_2Cr_2O_7$, both in BHK and HEp cultures (Fig. 1A) and with $10^{-4}$M concentration only in HEp cultures (Fig. 1B), showing that in the latter cell system the transition from the stimulation to the inhibition phase of permeases is advanced.

BHK survival curve to $K_2Cr_2O_7$ (Fig. 3) indicates a multi-hit mechanism of cell inactivation similar to that observed by treating yeast cells (Bonatti and Abbondandolo, personal communication) and corresponds to the classical survival-curve shapes, namely an initial, rather pronounced shoulder followed by a linearly logarithmic decline in the number of survivors as the dose increases. If the straight portion of the curve is extrapolated back to the axis, the intersection occurs close to 10, a form of behaviour which would be exhibited if 10 independent, Cr induced events were required for cell death. It must be stressed that no threshold dose can be inferred from survival curves to $K_2Cr_2O_7$, and that the extrapolation value is too low to suggest an interaction of Cr with the replication enzymes (DNA polymerases) as the more relevant inactivation mechanism. The number of such molecules in the mammalian cell greatly exceeds the hit number extrapolated on survival curves (Bollum,

1975). Moreover, an independent, simultaneous inactivation of transcription and translation enzymatic systems would be required to explain the concomitant inhibition of RNA and protein syntheses by $K_2Cr_2O_7$. If on the other hand the DNA molecule is the target for the action of Cr on cell survival, it is likely that a few hits are sufficient to impair its replication capacity. The interaction between DNA and Cr could account also for a secondary inhibition of RNA and protein syntheses (Bianchi *et al.*, 1977).

With $CrCl_3$ an irreversible inhibition of DNA synthesis is observed only after 2–4 h treatments with $10^{-2}$M concentrations (Fig. 4) whereas a comparable effect is obtained with 100 times lower $K_2Cr_2O_7$ concentrations (Fig. 2). The stronger cytotoxic activity of $K_2Cr_2O_7$ is even more evident when the reduction of BHK cell survival is measured: comparable effects are induced by $K_2Cr_2O_7$ concentrations 1000 times lower than $CrCl_3$ concentrations (Table II).

It is generally accepted that only $Cr^{6+}$ penetrates through the plasma membrane, being reduced to $Cr^{3+}$ inside the cell and firmly bound to cell components (Grogan, 1958; Mertz, 1969). Transport of $Cr^{6+}$ in the form of chromate ion is very effective, its rate and therefore the final intracellular Cr concentration depending on the extracellular chromate levels (Vallee, 1969). Mechanisms for $Cr^{3+}$ transport are presently unknown; anyhow $Cr^{3+}$ entrance into the cells is deemed to be limited if not completely absent (Feldman *et al.*, 1969; Mertz, 1969; Polak *et al.*, 1973). However, significant amounts of $Cr^{3+}$ are detected in BHK cells after treatment with $10^{-3}$M $CrCl_3$, and are about 20 times lower than those observed after treatment with the same concentration of $K_2Cr_2O_7$ (Table V). Therefore, the much lower effectiveness of $CrCl_3$ on BHK macromolecular syntheses and cell survival cannot be entirely related to a lower $Cr^3$ uptake, but seem to depend also on the stability of $Cr^{3+}$ chelates and coordination complexes which are formed in the treatment solutions and which are characterized by a very low exchange rate with biological ligands (Mertz, 1969).

A marked difference in the cytotoxic activity of $Cr^{6+}$ and $Cr^{3+}$ compounds has been observed also in *Salmonella* (Petrilli and De Flora, 1977) and in mouse cells (Raffetto *et al.*, 1977).

When BHK cells are treated with $10^{-2}$M $CrCl_3$, DNA and RNA become partially resistant to hydrolysis with PCA, an effect which is not observed with equimolar $K_2Cr_2O_7$ concentrations (Table III). Resistance of nucleic acids to hydrolysis with trichloroacetic acid has been described after treatment of animal tissues with $Cr^{6+}$ salts, and attributed to the interaction of nucleic acids with $Cr^3$ reduced inside the cells (Herrmann and Speck, 1954). The altered extractability of nucleic acids is probably due to the stabilization of their tertiary structure by $Cr^{3+}$, according to the modification of some physico-chemical properties of purified RNA in the presence of $CrCl_3$ (Huff *et al.*, 1964).

A stabilization of the DNA double helix by $Cr^{3+}$ is supported also by UV absorption spectra of nucleic acids purified from BHK cells treated with $CrCl_3$ (Tamino, 1977), by X-ray diffraction (Danchin, 1975) and NMR studies (Eisinger, Shulman and Szymanski, 1962) of DNA-$Cr^{3+}$ complexes. An interaction with DNA could represent also the final effect of $K_2Cr_2O_7$ after it is reduced to $Cr^{3+}$ inside the cells.

This work was supported by a grant from the National Research Council of Italy (C.N.R.).

### REFERENCES

Bianchi, V., Buttignol, M. & Levis, A. G. (1977) Specific Effects of $Cr^{b+}$ on Nucleic Acid Synthesis and Nucleoside Uptake in Hamster Cells (BHK Line). *Atti Ass. Genet. It.*, **22**, 65.

Bonatti, S., Meini, M. & Abbondandolo, A. (1976) Genetic Effects of Potassium Dichromate. *Mut. Res.*, **38**, 147.

Bollum, F. J. (1975) Mammalian DNA Polymerases. *Prog. Nucl. Acid Res. mol. Biol.*, **15**, 109.

Browning, E. (1969) Chromium. In *Toxicity of Industrial Metals.* London: Butterworths. p. 119.

DANCHIN, A. (1975) Labeling of Biological Macromulecules with Covalent Analogs of Magnesium. II. Features of the Chromic Cr(III) Ion. *Biochimie*, **57**, 875.

EISINGER, J., SHULMAN, R. C. & SZYMANSKI, B. M. (1962) Transition Metal Binding in DNA Solutions *J. Chem. Phys.*, **36**, 1721.

FELDMAN, F. Y., KNOBLOCK, E. C. & PURDY, W. C. (1967) The Determination of Chromium in Biological Materials by Atomic Absorption Spectroscopy. *Anal. Clin. Acta*, **38**, 489.

FRADKIN, A., JANOFF, A., LANE, B. P. & KUSCHNER, M. (1975) *In vitro* Transformation of BHK 21 Cells Grown in the Presence of Calcium Chromate. *Cancer Res.*, **35**, 1058.

FURST, A. & HARO, R. T. (1969) A Survey of Metal Carcinogenesis. *Prog. exp. Tumor Res.*, **12**, 102.

GLÄSS, E. (1956) Untersuchungen über die Einwirkung von Schwermetallsalzen auf die Wurzelspitzenmitose von *Vicia faba*. *Z. Bot.*, **44**, 1.

GROGAN, C. H. (1957) Experimental Studies in Metal Cancerogenesis. VIII. On the Etiological Factor in Chromate Cancer. *Cancer, N.Y.*, **10**, 625.

GROGAN, C. H. (1958) Experimental Studies in Metal Cancerogenesis. XI. On the Penetration of Chromium into the Cell Nucleus. *Cancer, N.Y.*, **11**, 1195.

HERRMANN, H. & SPECK, L. B. (1954) Interaction of Chromate with Nucleic Acids in Tissues. *Science, N.Y.*, **119**, 221.

HUEPER, W. C. (1958) Experimental Studies in Metal Cancerogenesis. X. Cancerogenic Effects of Roasted Chromite Ore Deposited in Muscle Tissue and Pleural Cavity of Rats. *Arch. ind. Health*, **18**, 284.

HUEPER, W. C. (1961) Environmental Carcinogenesis and Cancers. *Cancer Res.*, **21**, 842.

HUFF, J. W., SASTRY, K. S., GORDON, M. P. & WACKER, W. E. C. (1964) The Action of Metal Ions on Tobacco Mosaic Virus Ribonucleic Acid. *Biochemistry*, **3**, 501.

INTERNATIONAL AGENCY FOR RESEARCH ON CANCER (1973) Chromium and Inorganic Chromium Compounds. In *Monographs on the Evaluation of Carcinogenic Risk of Chemicals to Man*, **2**. Lyon: I.A.R.C. p. 100.

LEVIS, A. G. & BUTTIGNOL, M. (1977) Effects of Potassium Dichromate on DNA Synthesis in Hamster Fibroblasts. *Br. J. Cancer.*, **35**, 496.

LEVIS, A. G., BUTTIGNOL, M. & VETTORATO, L. (1977) Inhibition of DNA Synthesis in BHK Fibroblasts Treated *in vitro* with Potassium Dichromate. *Experientia*, **33**, 82.

LEVIS, A. G., BUTTIGNOL, M., BIANCHI, V. & SPONZA, G. (1978) Effects of Potassium Dichromate on Nucleic Acid and Protein Syntheses and on Precursor Uptake in BHK Fibroblasts. *Cancer Res.*, **38**, 110

MAJONE, F. (1977) Effects of Potassium Dichromate on Mitosis of Cultured Mammalian Cells. *Caryologia*, (In press).

MALTONI, C. (1974) Occupational Carcinogenesis. *Excerpta Med. Int. Cong. Ser.*, **322**, 19.

MALTONI, C. (1976) Predictive Value of Carcinogenesis Bioassays. *Ann. N.Y. Acad. Sci.*, **271**, 431.

MERTZ, W. (1969) Chromium Occurrence and Function in Biological Systems. *Physiol. Rev.*, **49**, 163.

MILLER, E. C. & MILLER, J. A. (1971) The Mutagenicity of Chemical Carcinogens: Correlations, Problems, and Interpretation. In *Chemical Mutagens*. Vol. 1. Ed. A. Hollaender. New York: Plenum Press. p. 83.

NISHIOKA, H. (1975) Mutagenic Activities of Metal Compounds in Bacteria. *Mut. Res.*, **31**, 185.

PAYNE, W. W. (1960) The Role of Roasted Chromite Ore in Production of Cancer. *Arch. environm. Health*, **1**, 20.

PETRILLI, F. L. & DE FLORA, S. (1977) Toxicity and Mutagenicity of Hexavalent Chromium on *Salmonella typhimurium*. *Appl. environm. Microb.*, **33**, 805.

POLAK, L., TURK, J. L. & FREY, J. R. (1973) Studies on Contact Hypersensitivity to Chromium Compounds. *Progr. Allergy*, **17**, 145.

RAFFETTO, G., PARODI, S., DE FERRARI, M., PARODI, C., TROIANO, R. & BRAMBILLA, G. (1977) Direct Interaction with Cellular Targets as the Mechanism for Chromium Carcinogenesis. *Eur. J. Cancer* (submitted).

SCHOENTAL, R. (1975) Chromium Carcinogenesis, Formation of Epoxy-aldehydes and Tanning. *Br. J. Cancer*, **32**, 403.

SIROVER, M. A. & LOEB, L. A. (1976) Infidelity of DNA Synthesis *In vitro*: Screening for Potential Metal Mutagens or Carcinogens. *Science, N.Y.*, **194**, 1434.

TAMARO, M., BANFI, E., VENTURINI, S. & MONTI BRAGADIN, C. (1976) I Composti del Cromo Esavalente Sono Mutageni per i Batteri. *Atti 17th Congr. Naz. Soc. It. Microbiol.* p. 411.

TAMINO, G. (1977) Interactions of Chromium with Nucleic Acids of Mammalian Cells. *Atti Ass. Genet. It.*, **22**, 69.

VALLEE, M. (1969) Le Système de Transport de Sulfate chez *Chlorella pyrenoidosa* et sa Régulation. IV. Etudes avec l'Ion Chromate. *Biochim. biophys. Acta*, **173**, 486.

VENITT, S. & LEVY, L. S. (1974) Mutagenicity of Chromates in Bacteria and its Relevance to Chromate Carcinogenesis. *Nature, Lond.*, **250**, 493.